# Chemo-enzymatic total synthesis: current approaches toward the integration of chemical and enzymatic transformations

**DOI:** 10.3762/bjoc.20.151

**Published:** 2024-07-23

**Authors:** Ryo Tanifuji, Hiroki Oguri

**Affiliations:** 1 Department of Chemistry, Graduate School of Science, The University of Tokyo, 7-3-1 Hongo, Bunkyo-ku, Tokyo 113-0033, Japan

**Keywords:** chemo-enzymatic synthesis, late-stage modification, reactive biosynthetic intermediate, regio- and stereoselective (macro)cyclization, total synthesis

## Abstract

A steadily increasing number of reports have been published on chemo-enzymatic synthesis methods that integrate biosynthetic enzymatic transformations with chemical conversions. This review focuses on the total synthesis of natural products and classifies the enzymatic reactions into three categories. The total synthesis of five natural products: cotylenol, trichodimerol, chalcomoracin, tylactone, and saframycin A, as well as their analogs, is outlined with an emphasis on comparing these chemo-enzymatic syntheses with the corresponding natural biosynthetic pathways.

## Introduction

**Scheme 1:**
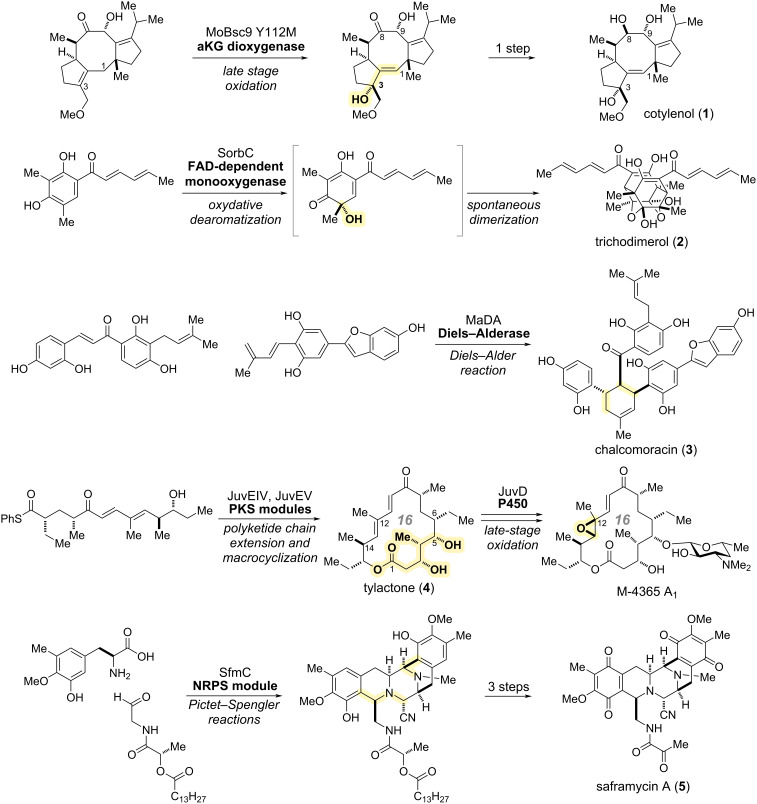
Targeted natural products and key enzymatic transformations in the chemo-enzymatic total syntheses featured in this review. Enzymatically formed bonds or units are highlighted in yellow.

Naturally occurring organic compounds with potent biological activities continue to be compelling lead candidates for drug discovery, with advancements in their synthesis and supply techniques progressing rapidly. Of particular note is the progress made in the "chemo-enzymatic approach" merging efficient enzymatic synthesis – traditionally employed in the biosynthesis of natural products by microorganisms and plants – with precise chemical synthesis conducted by chemists. Chemo-enzymatic total syntheses reported recently fall into three main categories based on the purpose for using an enzyme or at what stage in a synthesis the enzyme is employed: 1) regio- and stereoselective late-stage functionalization of core scaffolds, 2) in situ generation of highly reactive intermediates, and 3) the one-step construction of macrocyclic or fused multicyclic scaffolds via regio- and stereoselective cyclization reactions. This review aims to provide an overview of these approaches and parallel comparisons with original biosynthetic pathways by highlighting five examples of chemo-enzymatic total syntheses of natural products reported since 2017. The examples are the synthesis of cotylenol (**1**), trichodimerol (**2**), chalcomoracin (**3**), tylactone (**4**), and saframycin A (**5**), as well as a number of analogues of these natural products (Scheme 1). The overview of all five natural products begins with a description of the well-studied biosynthetic strategies evolved by microorganisms and plants. Biosynthetic pathways are described with a focus on the biosynthetic intermediates and enzymatic transformations that enable cascade reactions and pinpoint modifications. This focus highlights how these biosynthetic pathways are applicable to the development of streamlined chemo-enzymatic synthesis processes. The discussion will also encompass the design of biosynthetic intermediates and their analogs to achieve chemo-enzymatic total syntheses. Given our emphasis on natural products, this review does not cover the exquisite synthetic approaches involving biocatalysts for small-molecule pharmaceuticals. To gain a more comprehensive understanding of the chemo-enzymatic synthetic approach, we refer the reader to recent excellent reviews that provide multiple perspectives on the topic [1–10].

## Review

### Late-stage oxidative transformations of natural product scaffolds: cotylenol and brassicicenes

**Scheme 2:**
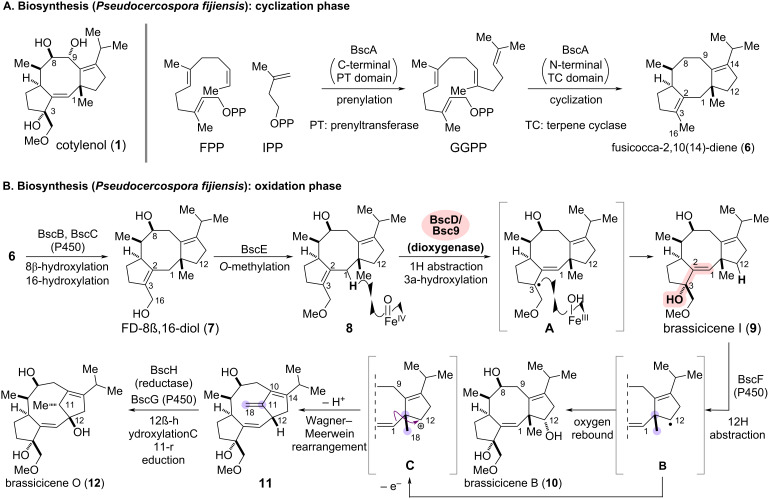
Biosynthetic pathway to brassicicenes in *Pseudocercospora fijiensis* [14]. (A) Cyclization phase catalyzed by BscA. (B) Oxidation phase and scaffold rearrangements by modification enzymes.

Chemo-enzymatic synthesis, featuring the late-stage enzymatic oxidation of chemically synthesized intricate scaffolds are attracting increasing attention. A collaboration between Stoltz and Arnold led to the pioneering accomplishment in the total synthesis of nigelladine A by exploiting P450 enzymes engineered through directed evolution [11]. P450 catalysis during the oxidation phase enabled the total synthesis of mitrephorone A [12], chevalone A [13], polysin [14], excolide B [15], and gedunin [16]. Fe(II)/2OG-dependent dioxygenases, such as FtmOx1, were employed as versatile catalysts in the synthesis of 13-oxoverruculogen [17]. The use of prenyltransferase NotF and flavin monooxygenase BvnB allowed the synthesis of eurotiumin A [18]. Although this review cannot cover the extensive recent progress in this type of chemo-enzymatic approach, we here provide an overview of the very recently reported chemo-enzymatic hybrid syntheses of cotylenol (**1**) and brassicicenes [19]. The key oxidative allylic rearrangement was conducted enzymatically, while the skeletal rearrangement originally mediated by P450 enzymes in the biosynthetic pathway was achieved through chemical transformation. Hence, this strategy can be considered a remarkable example of utilizing the complementarity between chemical and enzymatic transformations (Scheme 1 and Scheme 2).

The cotylenin and fusicoccin families comprise structurally related diterpene glucosides with a 5/8/5 fused tricyclic aglycon and a sugar moiety linked through the C9 hydroxy group of the aglycon. Cotylenin exhibits promising anticancer activity, and its diterpene aglycon, cotylenol (**1**), was isolated from the filamentous fungi such as *Phomopsis amygdali* and *Cladosporium* sp. 501-7W (Scheme 2A) [20–22]. Brassicicenes, differing in oxidation levels from **1**, have been isolated from *Alternaria brassicicola*, a phytopathogenic fungus that causes dark leaf spots in *Brassica* species. The biosynthesis process of brassicicenes was primarily elucidated through in vitro enzymatic transformations and heterologous expression using *Aspergillus oryzae*. The proposed biosynthetic pathway of brassicicenes in *Pseudocercospora fijiensis* is outlined based on an investigation by Oikawa and co-workers [23].

The biosynthesis begins with the conversion of farnesyl pyrophosphate (FPP) and isopentenyl pyrophosphate (IPP) to geranylgeranyl pyrophosphate (GGPP), catalyzed by the prenyltransferase (PT) domain located at the C-terminus of BscA (Scheme 2A). Subsequently, the terpene cyclase (TC) domain at the N-terminal of BscA generates fusicocca-2,10(14)-diene (**6**), which bears the common 5/8/5 fused tricyclic scaffold common to this natural products family.

Sequential oxidative conversions of scaffold **6** yield a series of intermediates and natural products, including cotylenol (**1**) and brassicicenes I and B (**9** and **10**), as well as brassicicene O (**12**), which possesses a distinct scaffold resulting from a skeletal rearrangement. To the core scaffold **6**, the P450 enzymes, BscB and BscC, introduce hydroxy groups at C8 and C16 to produce FD-8β,16-diol (**7**), and BscE-catalyzed *O*-methylation generates the putative intermediate **8**. The subsequent oxidative allylic rearrangement (**8**→**9**), catalyzed by the nonheme iron(II) and 2-oxoglutarate (Fe(II)/2OG)-dependent dioxygenase BscD, was a key step toward developing a chemo-enzymatic synthetic process. Presumably, the reactive iron(IV)-oxo species in dioxygenase BscD abstracts an allylic hydrogen at C1 and generates intermediate **A**. Subsequent α-face-selective hydroxylation of the resulting allylic radical at the C3 position would yield brassicicene I (**9**). As a pioneering investigation to elucidate the mechanism of this essentially identical allylic oxidations by Fe(II)/2OG-dependent dioxygenase, Dairi and co-workers conducted in vitro enzymatic conversions with the homologous enzyme Bsc9, derived from *Alternaria brassicicola* ATCC96836 [24].

The P450 enzyme BscF is responsible for regioselective abstraction of a hydrogen at C12 and subsequent diastereoselective hydroxylation of the radical intermediate **B** to produce brassicicene B (**10**). Meanwhile, further single-electron oxidation of the intermediate **B** would trigger a Wagner–Meerwein-type skeletal rearrangement, providing the distinct skeleton of **11** via carbocation **C**. This rearrangement involves the preferential migration of an alkenyl group in **C** to the carbocation, followed by deprotonation at C18 to form an *exo*-olefin. β-face-selective hydroxylation at C12 in **11** by the P450 enzyme BscG, and subsequent reduction of the exomethylene at C11–C18 catalyzed by BscH yield brassicicene O (**12**).

**Scheme 3:**
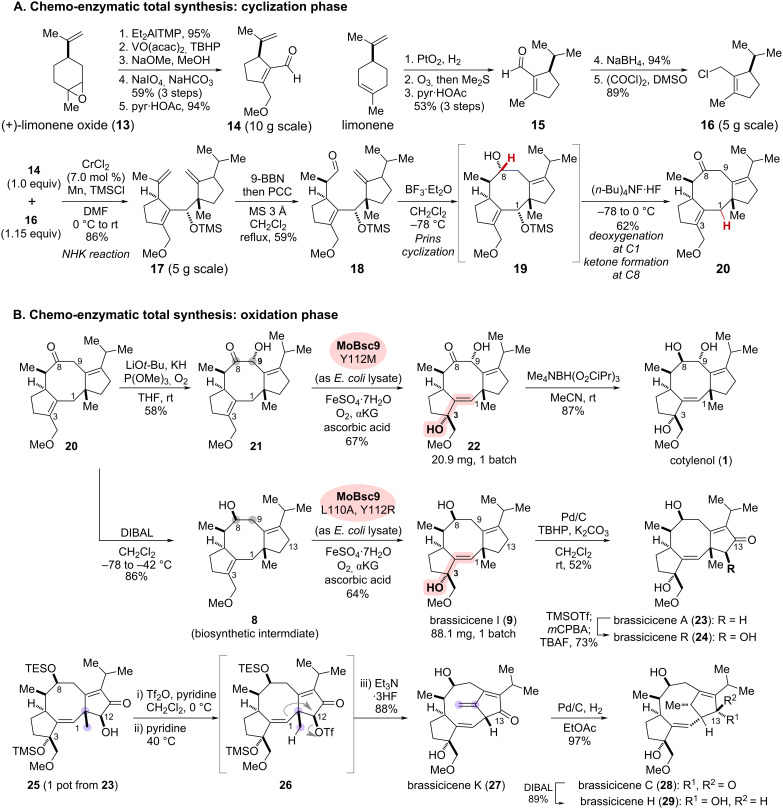
Chemo-enzymatic total synthesis of cotylenol (**1**) and brassicicenes. (A) Chemical cyclization phase. (B) Chemical and enzymatic oxidation phase.

Renata and co-workers successfully accomplished the chemoenzymatic total syntheses of cotylenol (**1**) and nine brassicicenes (Scheme 3) [19]. In the cyclization phase, a suitably functionalized 5/8/5 tricyclic scaffold was produced through scalable chemical synthesis. Subsequently, in vitro enzymatic oxidative functionalizations were carried out during the oxidation stage. The exploration of Bsc9 homologs and directed evolution expanded the scope of substrates of the dioxygenase beyond the natural biosynthetic intermediate **8** to its analogs **21**, enabling the chemo-enzymatic total synthesis of **1**.

The chemical synthesis commenced with the preparation of two fragments and their subsequent coupling to assemble the core 5/8/5 tricyclic scaffold (Scheme 3A). The left-half fragment, aldehyde **14**, was synthesized on a decagram scale in five steps from (+)-limonene oxide (**13**), involving epoxide manipulation, oxidative cleavage, and intramolecular aldol condensation. Similarly, the right-half fragment, allyl chloride **16**, was synthesized from limonene in five steps. Site-selective hydrogenation, oxidative cleavage, and intramolecular cyclization provided **15**, followed by functional group manipulation to yield **16**. The two segments **14** and **16** were assembled by Nozaki–Hiyama–Kishi (NHK) coupling while controlling the regio- and diastereoselectivities to afford intermediate **17** [25]. Site-selective hydroboration and subsequent oxidation yielded aldehyde **18**, a precursor for the intramolecular ring closure of the eight-membered ring. Upon treatment of **18** with BF_3_·Et_2_O, diastereoselective Prins cyclization of **18** proceeded to generate secondary alcohol **19**. Subsequent one-pot treatment with (*n*-Bu)_4_NF·HF resulted in unexpected conversions, including the formation of an allylic carbocation at C1, followed by transannular hydride transfer from C8 to afford ketone **20** in 62% yield.

With the 5/8/5 tricyclic scaffold **20** in hand, site- and diastereocontrolled C9 hydroxylation of **20** produced a substrate **21** for the enzymatic conversions, commencing with the oxidation phase (Scheme 3B). As shown in Scheme 2B, the two cognate biosynthetic enzymes, BscD and Bsc9, are responsible for the oxidative allylic rearrangement (**8**→**9**). While soluble BscD was not obtained, the overexpressed dioxygenase Bsc9 enabled to catalyze the regio- and diastereocontrolled hydroxylation at C3, along with transposition of the double bond, to form the desired product **22** in approximately 20% yield with 50% conversion. To enhance efficiency of the enzymatic oxidation, Renata and co-workers conducted two approaches: homolog screening and enzyme engineering. A Genome Neighborhood Diagram (GND) analysis [26] of dioxygenase Bsc9 identified five homologs, including a homolog MoBsc9 derived from *Magnaporthe oryzae,* which shares 54% sequence identity with Bsc9 and was found to be suitable for the oxidative allylic rearrangement (**21**→**22**). Further directed evolution using site-saturation mutagenesis targeting the putative active sites L110 and Y112, led to the variant MoBsc9 Y112M, which substantially improved the enzymatic conversion into **22**, achieving an isolated yield of up to 67%. Diastereoselective reduction of the C8 ketone was then achieved using the protocol of Nakada and co-workers [27], enabling the chemo-enzymatic total synthesis of cotylenol (**1**).

Similarly, diastereoselective reduction of the C8 ketone in **20** yielded the biosynthetic intermediate **8** for brassicicenes. Although substrate **8** has a different oxidation state at C8 and lacks the C9 secondary alcohol of **21**, the utilization of another variant, MoBsc9 L110A, Y112R, generated through the directed evolution of MoBsc9, facilitated the optimal conversion in the corresponding oxidative allylic rearrangement to afford brassicicene I (**9**) in 64% yield. Further palladium-catalyzed allylic oxidation with the incorporation of a ketone at the C13 position yielded brassicicene A (**23**). After conversion of **23** into the corresponding silyl enol ether, Rubottom oxidation allowed completion of the total synthesis of brassicicene R (**24**).

As an effort to explore the biomimetic rearrangement, analogous to the biosynthetic conversion of **9** into **11** (Scheme 2B), an α-hydroxylated ketone **25** with suitable protections of the C3 and C8 hydroxy groups was synthesized from **23** in one pot. Conversion of the secondary alcohol in **25** into triflate **26** enabled the alkenyl shift from C1 to C12 followed by deprotonation to form exomethylene. Subsequent in situ removal of the silyl protecting groups led to the total synthesis of brassicicene K (**27**). Thus, the biosynthetic proposal featuring the Wagner–Meerwein-type skeletal rearrangement (**9**→**11**) through the catalysis of P450 enzyme BscF was successfully emulated by the chemical conversion.

Site- and diastereocontrolled hydrogenation of the resulting exomethylene in **27** yielded brassicicene C (**28**). Further diastereoselective reduction of the C13 ketone completed the total synthesis of brassicicene H (**29**). By integrating the convergent and scalable chemical synthesis of the 5/8/5 scaffold with enzymatic regio- and stereoselective oxidative conversions, Renata and co-workers successfully achieved the collective chemo-enzymatic total synthesis of ten natural diterpenes, five with a 5/8/5 tricyclic skeleton and five with a rearranged scaffold including brassicicenes **27**–**29**.

### In situ generation of highly reactive intermediates: trichodimerol and the bisorbicillinoid family

**Scheme 4:**
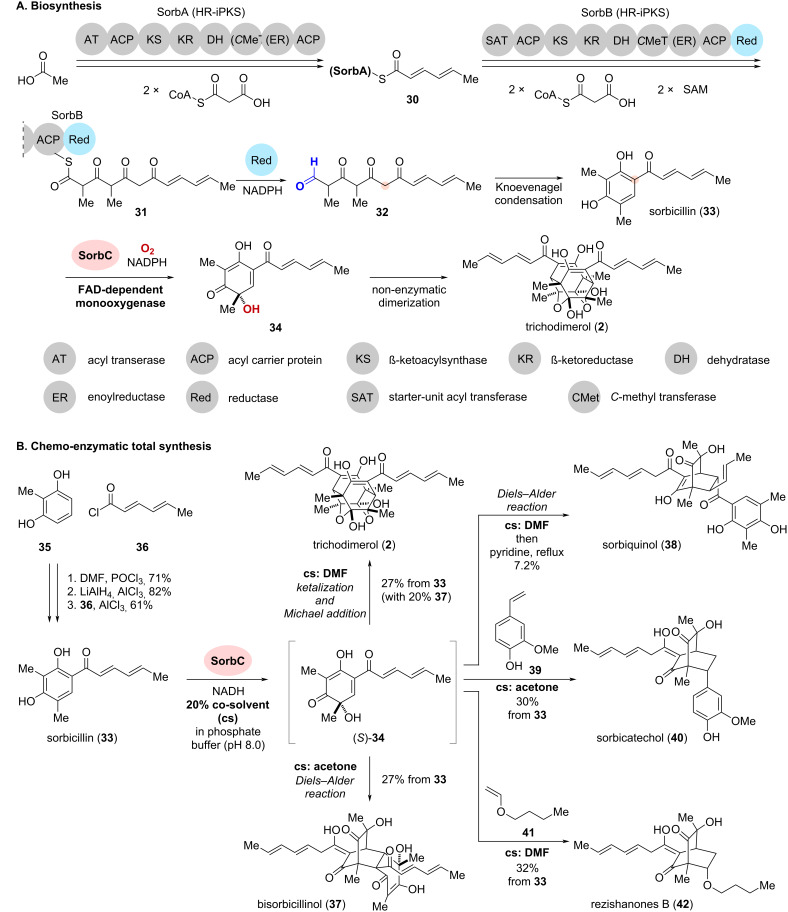
(A) Biosynthetic pathway for trichodimerol (**2**) in *Penicillium chrysogenum*. (B) Chemo-enzymatic total synthesis of **2** and bisorbicillinoids utilizing an FAD-dependent monooxygenase SorbC.

The bisorbicillinoid family, isolated from fungi such as *Penicillium chrysogenum*, has dimeric intricate scaffolds, as exemplified by trichodimerol (**2**) (Scheme 4) [28–29]*.* The biosynthetic pathway of these natural products involves late-stage skeletal diversification triggered by enzymatic oxidative dearomatization (Scheme 4A). Specifically, FAD-dependent monooxygenase catalyzes the oxidative dearomatization of the aromatic intermediate, leading to subsequent homo- and hetero-dimerization processes. The mechanism was initially postulated by Dreiding [30], and supported through isotope-labelling studies conducted by Abe and co-workers [31–33]. Subsequently, Cox et al. have elucidated the key enzyme SorbC responsible for the dearomatization process [34–35].

Scheme 4A illustrates the biosynthetic pathway as proposed by Cox, based on bioinformatic analyses of polyketide synthase (PKS) modules and in vitro studies. Initially, highly reducing iterative polyketide synthase (HR-iPKS) SorbA forms the thioester **30** on its acyl carrier protein (ACP) domain from acetate and two units of malonyl-CoA. Subsequently, the downstream HR-iPKS module, SorbB, catalyzes both the extension of polyketide chain from **30** using two additional malonyl-CoAs and *C*-methylations exploiting two units of SAM (*S*-adenosyl methionine) to generate thioester **31** on the ACP domain. Then, the reduction (Red) domain located at the C-terminus of SorbB catalyzes conversion of thioester **31** using NADPH to liberate aldehyde **32**, and subsequent Knoevenagel condensation-type cyclization leads to sorbicillin (**33**).

The FAD-dependent monooxygenase SorbC, utilized in the chemo-enzymatic total synthesis of **2**, catalyzes the oxidative dearomatization of **33** via enantioselective hydroxylation using molecular oxygen and generates cyclohexadienone **34**. As demonstrated by Corey [36] and Nicolaou [37], highly reactive intermediate **34** likely dimerizes non-enzymatically through stepwise reactions involving (1) an initial intermolecular Michael addition, (2) a second intramolecular Michael reaction forming a head to tail formal [4 + 4] cycloadduct, and (3) sequential hemiketal formations to furnish the densely-functionalized and complicated pentacyclic scaffold of trichodimerol (**2**). Alternatively, the 2,4-cyclohexadienone moiety of **34** undergoes intermolecular Diels–Alder reactions with **34** or other dienophiles, leading to a diverse array of bisorbicillinoids. Based on the crystal structure of TropB, a homologous enzyme of SorbC, Narayan and co-workers have intensively elucidated that the detailed mechanisms of the oxidative dearomatization catalyzed by the FAD-dependent monooxygenases [38–39].

Gulder and co-workers achieved the chemo-enzymatic total synthesis of bisorbicillinoids family members by merging chemical synthesis and the enzymatic oxidative dearomatization (Scheme 4B) [40–41]. The SorbC-catalyzed enzymatic oxidation of chemically synthesized **33** generated the highly reactive intermediate **34** in aqueous solvents under mild conditions [37,42]. In this process, the co-solvent (cs) allowed control of the dimerization modes via either Michael addition or Diels–Alder reactions, facilitating the systematic total synthesis of the bisorbicillinoid family.

Substrate **33** for SorbC-catalyzed enzymatic transformation was synthesized in 3 steps from phenol **35** via formylation and subsequent reduction to introduce a methyl group, followed by a Friedel–Crafts acylation with **36** (Scheme 4B) [39–40]. The in vitro enzymatic transformation of **33** by recombinant SorbC enantioselectively introduced a hydroxy group to generate the reactive intermediate (*S*)-**34** [34]. Cyclohexadienone (*S*)-**34** was relatively stable in aqueous reaction solvents, however, quenching the enzymatic reaction by the addition of organic solvents led to homo-dimerization. Based on these experimental results, Gulder and co-workers devised a strategy to control the dimerization modes by adjusting the polarity of the organic co-solvent to establish the divergent synthesis of dimeric scaffolds. Indeed, with 20% DMF in the SorbC-catalyzed enzymatic oxidative dearomatization, the Michael addition/ketalization cascades of generated (*S*)-**34** proceeded predominantly, achieving the chemoenzymatic total synthesis of trichodimerol (**2**) in 27% yield from **33**. In contrast, the use of acetone as the co-solvent resulted in the homodimerization of (*S*)-**34** via Diels–Alder reaction between the cyclohexadienone moieties, allowing the total synthesis of bisorbicillinol (**37**). Meanwhile, treatment of the resulting (*S*)-**34** under harsher conditions, such as reflux in pyridine, resulted in a distinct intermolecular Diels–Alder reaction between the cyclohexadienone moiety and the side chain of (*S*)-**34** enabling the first total synthesis of sorbiquinol (**38**). This chemo-enzymatic synthesis exploiting (*S*)-**34** generated through enzymatic transformation as a common and versatile intermediate, illustrates how judicious choice of the co-solvent can successfully diversify multicyclic complex scaffolds. The enhancement of organic solvent tolerance through enzyme mutagenesis should allow more precise control over scaffold-constructing reactions.

As with the related natural products, hybrid sorbicillinoids were also known to be biosynthesized through Diels–Alder reactions between diene **34** and non-sorbicillinoid-derived dienophiles [29]. Gulder and co-workers achieved the total synthesis of hybrid sorbicillinoids using the established chemo-enzymatic synthetic process, in addition to the synthesis of homodimers (Scheme 4B). Upon treatment of the extract of the enzymatic reaction mixture containing (*S*)-**34** with dienophile **39**, sorbicatechol (**40**) was obtained in 30% yield from **33**. The use of dienophile **41** instead of **39** led to the concise total synthesis of rezishanone B (**42**) in 32% yield. The same research group has also facilitated rapid access to bisorbicillinoid analogs, by using either various dienophiles or non-natural type substrates of SorbC [43].

Almost concurrently, Narayan and co-workers substantially expanded the application scope of synthetic methodologies by utilizing the FAD-dependent monooxygenase TropB, AzaH, and SorbC to catalyze oxidative dearomatization [38–39]. Through extensive exploration of substrate diversity, the research group achieved the synthesis of various dearomatized compounds, and the total synthesis of a member of the sorbicillinoid family.

### Enantioselective intermolecular Diels–Alder reaction to assemble core scaffolds: chalcomoracin and kuwanons

**Scheme 5:**
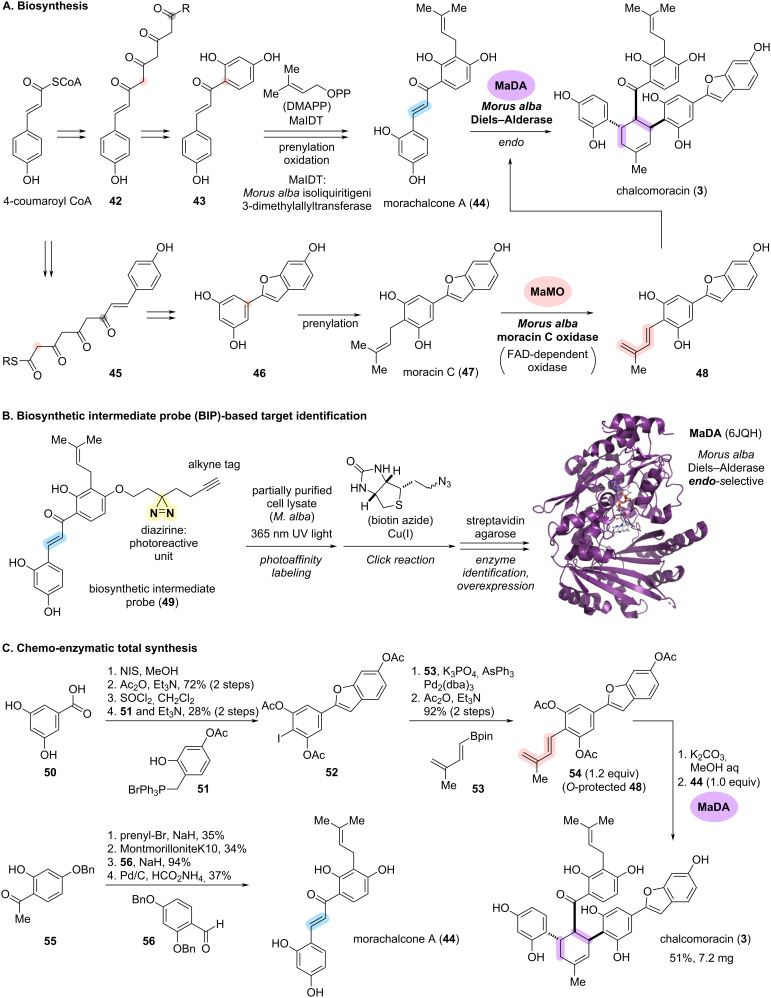
(A) Proposed biosynthetic pathway for chalcomoracin (**3**) in *Morus alba*. (B) Outline of the biosynthetic intermediate probe (BIP)-based target identification strategy. (C) Chemo-enzymatic total synthesis of **3** utilizing an identified Diels–Alderase, MaDA.

Chalcomoracin (**3**) and kuwanons bearing a highly substituted methyl cyclohexene core were isolated from plants of the *Moraceae* family as phytoalexins (Scheme 5) [44]. Since more than 160 related compounds have been discovered in an optically active form, Masamune, Takahashi, and colleagues postulated that the core scaffold is biosynthesized via Diels–Alder (DA) reactions [45]. In the 1980s, the research group led by Nomura supported this biosynthetic hypothesis by isotope labelling experiments using cell cultures of *Morus alba* [46–48]. Furthermore, the proposed DA reaction precursors to **3**, morachalcone A (**44**) and moracin C (**47**), were also isolated [47,49].

As shown in Scheme 5A, the proposed biosynthetic pathway of chalcomoracin (**3**) commence with the addition of three C2 units from the acetate pathway to 4-coumaroyl-CoA supplied from the shikimate pathway, leading to compound **42** [47]. Subsequent Claisen condensation, dehydration and aromatization provides chalcone **43**. Prenylation of the resultant aromatic ring of **43**, catalyzed by MaIDT (*Morus alba* isoliquiritigenin 3-dimethylallyltransferase), leads to morachalcone A (**44**) [50–51]. In parallel, benzofuran **46** was biosynthesized from 4-coumaroyl-CoA via thioester **45**. Further prenylation of phenol **46** yields moracin C (**47**). As the final steps in the biosynthesis of **3**, oxidation of the prenyl group of **47** to diene **48** and the subsequent DA reaction with dienophile **44** should theoretically be catalyzed by oxidase and Diels–Alderase, respectively. However, despite effort over several decades, these enzymes had not been discovered from *Morus alba*.

In 2020, Lei and co-workers successfully identified two key enzymes in the biosynthesis of **3**, MaMO (*M. alba* moracin C oxidase) and MaDA (*M. alba* Diels–Alderase), by developing a novel method termed “biosynthetic intermediate probe (BIP)-based target identification” (Scheme 5B) [52–54]. In addition, the utilization of MaDA allowed the chemo-enzymatic total synthesis of **3** and related natural products (Scheme 5C).

To identify the Diels–Alderase (MaDA), the research group initially demonstrated an in vivo enzymatic reaction by treating chemically synthesized **44** and **47** with cultured *M. alba* cells. The formation of both diene **48** and DA adduct **3** were observed, indicating that *M. alba* cells harbor the key enzymes MaMO and MaDA. Based on these results, synthetic analogs of intermediate **44** were designed and exposed to the *M. alba* cells to explore the substrate tolerance of MaDA for chemical probe design. After several attempts, Lei and co-workers designed and synthesized chemical probe **49** bearing a diazirine photoaffinity labelling unit with an alkyne tag on the phenolic hydroxy group of biosynthetic intermediate **44** (Scheme 5B). The treatment of fractionated cell cultures of *M. alba* with **49** was followed by irradiation with 365 nm light to generate reactive carbene from diazirine. This sequence allowed the formation of covalent bonds between the synthetic probe and binding proteins. The resulting mixture was subjected to a copper-catalyzed click reaction with biotin azide, which led to selective pull-down with streptavidin agarose and isolation of the probe–protein covalent complex. Proteomic analysis of the isolated proteins narrowed down the MaMO and MaDA candidates, including several berberine bridge enzyme (BBE)-like enzymes. This FAD-linked oxidase family is known to catalyze a variety of oxidative transformations critical for natural products biosynthesis [55]. Further transcriptome analysis of the candidate proteins led to the identification of two BBE-like enzymes, MaMO and MaDA, as key biosynthetic enzymes for **3**. These enzymes were subsequently overexpressed in insect cells and purified as soluble proteins, culminating in the elucidation of the X-ray crystallographic structure of MaDA (Scheme 5B, PDB: 6JQH).

With these key enzymes in hand, Lei and co-workers conducted a chemo-enzymatic total synthesis of **3** and related natural products utilizing the successfully overexpressed Diels–Alderase, MaDA (Scheme 5C). The chemical synthesis of **54**, tri-*O*-acetylated precursor of the diene component **48**, commenced from phenol **50**. Iodination and *O*-acetylations of **50** followed by coupling with phosphorus ylide **51** afforded aryl iodide **52**. Subsequent Suzuki–Miyaura coupling with boronic ester **53** and *O*-acetylation furnished **54**. The dienophile component, morachalcone A (**44**), was synthesized from phenol **55** in four steps including *O*-prenylation and subsequent Claisen rearrangement, aldol condensation with **56**, and deprotection. The key chemo-enzymatic conversions, in situ generation of **48** by deprotection of **54**, followed by treatment with **44** in the presence of purified Diels–Alderase MaDA, facilitated an *endo*-selective DA reaction and led to the concise total synthesis of chalcomoracin (**3**) in 51% yield.

**Scheme 6:**
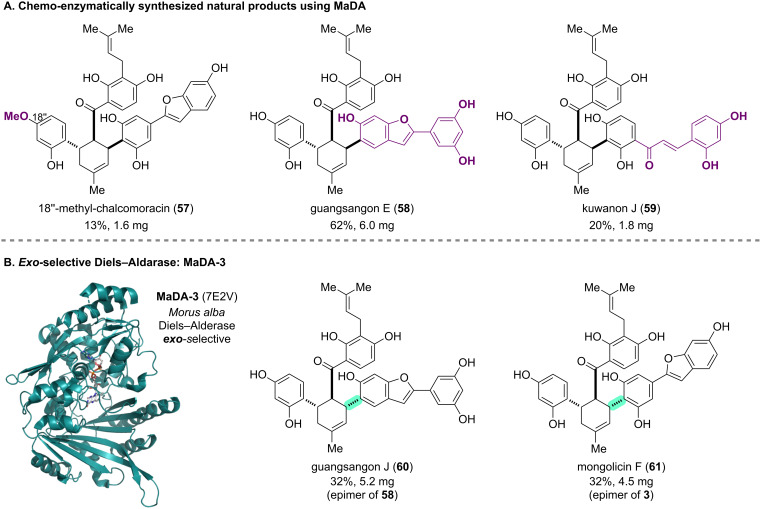
(A) Chemo-enzymatically synthesized natural products by using the originally identified MaDA. (B) MaDA homologue enzyme MaDA-3: X-ray crystal structure and synthesized natural products by leveraging this enzyme.

To achieve the systematic total synthesis of the natural product family sharing the highly substituted methyl cyclohexene moiety, Lei and co-workers synthesized analogs of biosynthetic intermediates **44** and **48**, and subjected them to the established chemo-enzymatic synthetic process (Scheme 6A) [56]. MaDA exhibited a relatively broad substrate tolerance, enabling the total synthesis of chiral natural products such as 18”-methyl-chalcomoracin (**57**), guangsangon E (**58**), and kuwanon J (**59**). Furthermore, the same research group identified a series of MaDA homologous enzymes in *Morus notabilis*, designated as MaDA-1–3, which showed distinct stereoselectivities in the Diels–Alder reactions (Scheme 6B). Notably, MaDA-3 exhibited high *exo*-selectivity (original MaDA: *endo*-selective) and enabled the enantioselective rapid total synthesis of guangsangon J (**60**) and mongolicin F (**61**), which are epimers of **58** and **3**, respectively. Comparative analysis of the X-ray crystallographic structures of MaDA (6JQH) and MaDA-3 (7E2V) provided insights into the mechanisms of different stereoselectivities in the Diels–Alder reactions [56]. In 2014, the same group led by Lei also reported the total synthesis of kuwanon J (**59**) and related natural products. In their earlier fully chemical approach, they used a chiral boron catalyst as a Lewis acid and achieved at best an *endo*/*exo* selectivity of 1.9:1 in a similar DA reaction. The use of Diels–Alderase in their recent work significantly improved the *endo*/*exo* selectivity under mild conditions in water, thereby highlighting the strengths of the chemo-enzymatic approach for synthesizing this family of natural products [57].

### Regioselective macrocyclization: tylactone and juvenimicins

**Scheme 7:**
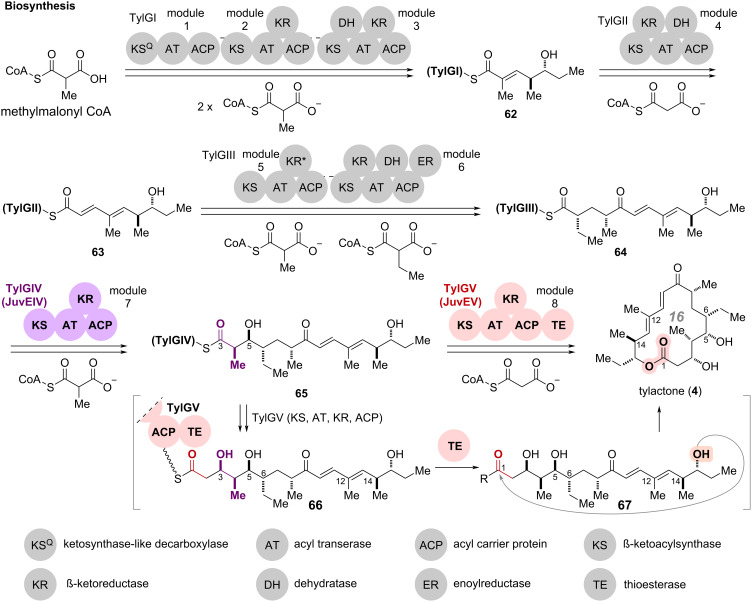
Proposed biosynthetic mechanism of tylactone (**4**) in *Streptomyces fradiae*.

**Scheme 8:**
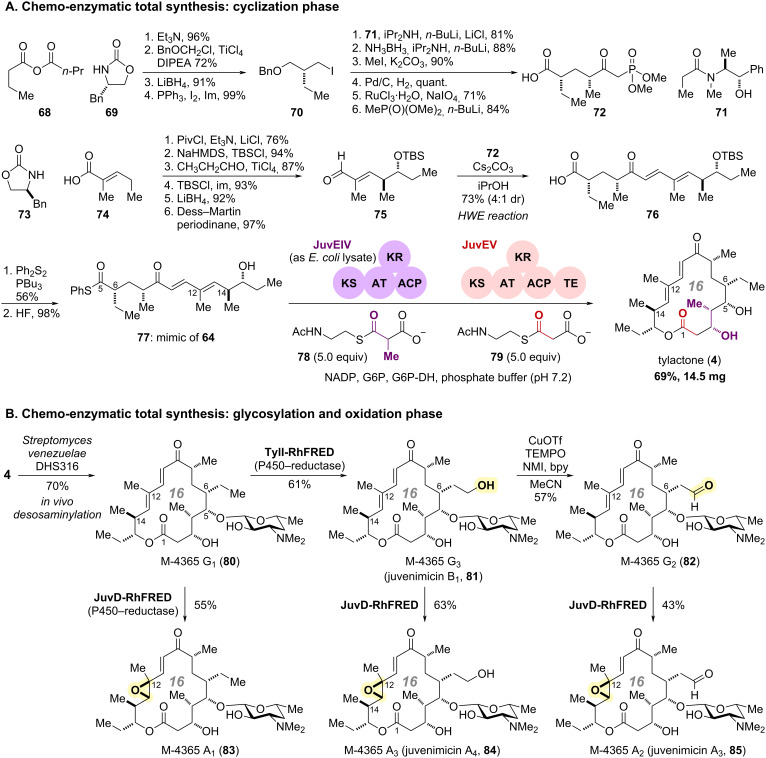
(A) Chemical synthesis and cascade enzymatic transformations of cyclization precursors. (B) Late-stage modifications of chemo-enzymatically synthesized aglycone **4** utilizing in vivo/in vitro enzymatic transformations and chemical conversions.

In the chemical synthesis of macrolides, macrolactone construction most generally involves the generation of activated esters from the corresponding carboxylic acids followed by intramolecular condensation. As a chemo-enzymatic complementary strategy, thioesterase (TE) domains of NRPS and PKS gene clusters have been utilized as enzymes for macrolactone formation to produce a wide range of natural/non-natural macrocyclic compounds [58–63]. In the field of chemo-enzymatic total synthesis utilizing polyketide synthase (PKS)-related macrocyclization enzymes, Xiang and co-workers recently reported the total synthesis of cylindrocyclophanes [64]. The cyanobacterial non-canonical enzyme CylK, discovered and characterized by Balskus and co-workers [65–67], facilitated an intramolecular Friedel–Crafts-type homo-dimerization and led to the efficient construction of the 22-membered paracyclophane scaffold. Of several sophisticated chemo-enzymatic strategies, this review highlights the total syntheses of tylactone (**4**) and juvenimicins (Scheme 7 and Scheme 8) [68]. This chemo-enzymatic total synthesis represents a pioneering approach, as it ingeniously integrates biosynthetic intermediate mimics, synthesized through multiple steps, into the biosynthetic pathway. A cascade of stereoselective carbon chain elongation and regioselective macrolactonization catalyzed by two massive PKS modules exemplifies a refined and innovative method in chemo-enzymatic synthesis.

Tylactone (**4**), a 16-membered macrolactone, was isolated from a *Streptomyces fradiae* mutant and characterized as the aglycone of the macrolide antibiotic tylosin (Scheme 7) [69]. Since the 1970s, the biosynthetic pathway of **4** has been investigated through isotope labelling and analysis of metabolites from *S. fradiae* mutants [70–73]. Heterologous production of **4** was also achieved by expression of elucidated biosynthetic gene cluster from *S. fradiae* in *Streptomyces venezuelae* [74–77].

In the proposed biosynthesis of **4** as illustrated in Scheme 7, five units of the type I polyketide synthase (PKS), TylGI–GV, comprising eight modules catalyze the polyketide chain extension reactions and a macrocyclization (Scheme 7) [78]. Initially, the acyltransferase (AT) domain of TylGI loads a methylmalonyl-CoA onto the acyl carrier protein (ACP) in module 1. The ketosynthase-like decarboxylase (KS^Q^) domain catalyzes the decarboxylation of the loaded methylmalonyl moiety, and subsequent modules 2 and 3 extend the carbon chain using two molecules of malonyl-CoA. The β-ketoreductase (KR) and dehydratase (DH) domains, sequentially catalyze the carbonyl reduction and dehydration of the extended polyketide chains to provide thioester **62** connecting to the ACP domain of module 3. TylGII then iterates similar conversions including carbon chain extension reaction, carbonyl reduction, and dehydration to generate the thioester **63** on its ACP domain. Module 5 of TylGIII further extends the carbon chain with incorporation of methylmalonyl-CoA and ethylmalonyl-CoA. Then the KR, DH, and enoylreductase (ER) domains of module 6 catalyze the formation and reduction of enone to furnish thioester **64** on the ACP domain.

Subsequent carbon chain extension and macrolactonization catalyzed by two PKS modules, TylGIV and TylGV, are pivotal enzymatic transformations for the chemo-enzymatic total synthesis of **4**. TylGIV catalyzes the carbon chain extension reaction using a methylmalonyl-CoA and the stereoselective reduction of the C5 carbonyl group to form thioester **65**. The subsequent PKS module TylGV introduces two more carbons from malonyl-CoA and reduces the carbonyl group at the C3 position to produce thioester **66** on the ACP domain. The thioesterase (TE) domain then catalyzes the release of **67** from the ACP domain and regioselective macrolactonization to furnish the 16-membered tylactone (**4**).

The research group led by Sherman accomplished the efficient chemo-enzymatic total synthesis of tylactone (**4**) and a series of M-4365 (juvenimicin) by merging consecutive enzymatic transformations by JuvEIV and JuvEV, homologous enzymes to TylGIV and TylGV (Scheme 8) [68]. Reconstruction of the JuvEIV–JuvEV-catalyzed cascade enzymatic conversions successfully furnished **4** in one pot from chemically synthesized thioester **77** mimicking biosynthetic intermediate **64**.

For the chemical synthesis of mimic **77**, the left-half segment **72** was prepared from mixed anhydride **68** (Scheme 8A). Condensation with chiral auxiliary **69** and subsequent diastereoselective alkylation, followed by reductive removal of the auxiliary and iodination of the resulting primary alcohol provided alkyl iodide **70** [78]. Subsequent six-step transformations including diastereoselective alkylation of (+)-pseudoephedrine derivative **71** [79] with the iodide **70**, sequential functional group manipulations, and installation of the β-ketophosphonate, provided the left-half segment **72** [80]. The right-half segment **75** was synthesized in six steps via condensation of **73** and **74**, followed by highly-diastereoselective vinylogous Mukaiyama aldol reaction and subsequent protecting group manipulations [81–83]. The separately synthesized left and right segments, **72** and **75**, were then assembled via Horner–Wadsworth–Emmons (HWE) olefination to give carboxylic acid **76** in a 4:1 diastereomeric ratio. Thioesterification and removal of the TBS group furnished the desired thioester **77** as a mimic of biosynthetic intermediate **64**.

With synthetic substrate **77** for enzymatic conversions in hand, Sherman and co-workers attempted to overexpress TylGIV and TylGV, however, they were faced with low expression levels of TylGV. To circumvent this problem, they employed the homologous enzymes JuvEIV and JuvEV from juvenimicin-producing *Micromonospora chalcea* subsp. *izumensis* [84–85]. The Juv gene cluster contained JuvEI–JuvEV with high homology to TylGI–TylGV, and P450 monooxygenases JuvC and JuvD for post-macrocyclization oxidative modifications.

Based on the successful cloning and high-level overexpression of the two key PKS modules, JuvEIV and JuvEV, cascade enzymatic conversions of **77** were conducted. The SNAC (*N*-acetylcysteamine) esters **78** and **79** were used as surrogates for methyl malonyl-CoA and malonyl-CoA in the enzymatic conversions. After careful and systematic optimization of the reaction conditions, such as the stoichiometry of the PKS modules and pH, along with the application of a NADPH recycling system, JuvEIV/JuvEV-catalyzed enzymatic conversions of **77**–**79** afforded tylactone (**4**) in one pot with 69% yield. By orchestrating the cooperation of two massive PKS modules (>150 kDa), this chemoenzymatic process enabled: (1) incorporation of fully synthetic hexaketide **77** as a substrate, (2) installation of the last four-carbon polyketide chain, (3) stereoselective introduction of three consecutive chiral centers, and (4) successful completion of the macrolactonization of the resulting octaketide to produce tylactone (**4**). This chemo-enzymatic process demonstrated highly efficient in vitro cascade transformations, underscoring the potential of integrating enzymatic catalysis with chemical synthesis [86].

By taking advantage of the chemo-enzymatically accessible **4**, Sherman and co-workers further implemented the systematic total synthesis of juvenimicins and the M-4365 series via enzymatic and chemical late-stage modifications (Scheme 8B) [68]. In vivo glycosylation utilizing *Streptomyces venezuelae* DHS316, developed by the same group, mediated the desosaminylation of **4** at C5 and afforded M-4365 G_1_ (**80**) in 70% yield [87]. For further oxidative modifications, they prepared the fusion proteins Tyll-RhFRED and JuvD-RhFRED, comprising P450 monooxygenases (TylI, JuvD) with the P450 RhF reductase domain (RhFRED) [88]. TylI-RhFRED facilitated the in vitro site-selective enzymatic hydroxylation of the C6 ethyl substituent in **80**, yielding M-4365 G_3_ (juvenimicin B_1_, **81**). Subsequent chemoselective oxidation of the resulting primary alcohol furnished M-4365 G_2_ (**82**) bearing an aldehyde at the C6 sidechain. Furthermore, the fusion protein, JuvD-RhFRED, enabled regio- and diastereoselective epoxidation of the C12 double bond of macrocycles **80**–**82**, culminating in the total synthesis of M-4365 A_1_ (**83**), M-4365 A_3_ (juvenimicin A_4_, **84**), and M-4365 A_2_ (juvenimicin A_3_, **85**), respectively.

### Iterative Pictet–Spengler cyclizations: saframycin A and jorunnamycin A

**Scheme 9:**
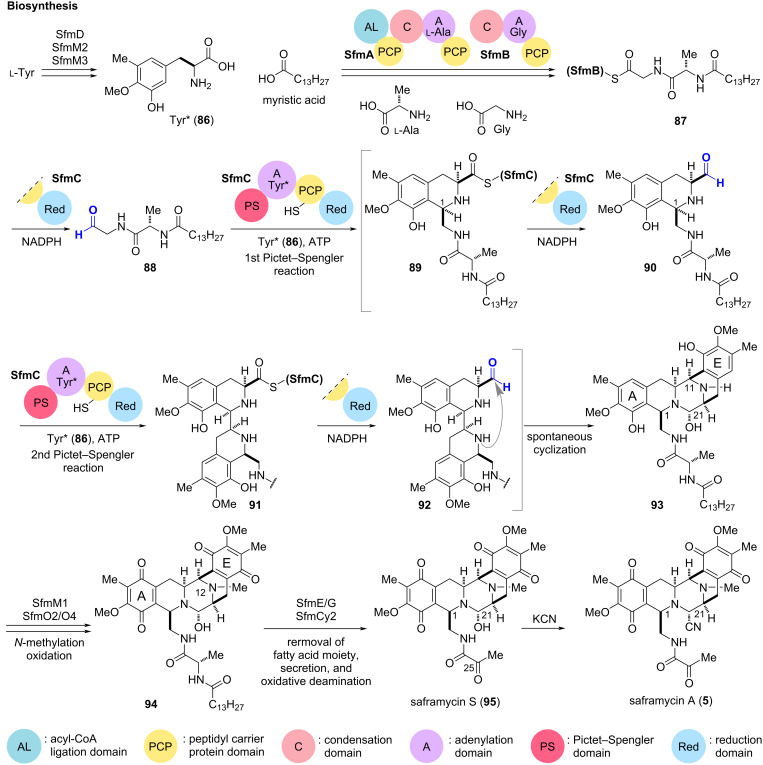
Proposed biosynthetic mechanism of saframycin A (**5**) in *Streptomyces lavendulae*.

Saframycin A (**5**) was isolated from *Streptomyces lavendulae*, and a number of related alkaloid families such as safracins and renieramycins have been identified from both soil and marine microorganisms [89–90]. These natural product families, known as bistetrahydroisoquinoline (THIQ) alkaloids, share a highly functionalized pentacyclic scaffold with different aromatic ring oxidation states and sidechain structures [90–93]. The biosynthetic mechanism of **5** has been extensively studied by gene disruption and reconstruction of in vitro enzymatic conversions (Scheme 9) [94]. The biosynthesis of **5** begins with the conversion of ʟ-tyrosine to tyrosine derivative **86** (Tyr*) by peroxygenase SfmD and the methyltransferases, SfmM2 and M3 [95–96]. Concurrently, two non-ribosomal peptide synthetase (NRPS) modules, SfmA and SfmB, catalyze the successive condensation of myristic acid, ʟ-alanine, and glycine to furnish thioester **87** on the peptidyl carrier protein (PCP) domain of SfmB [94,97]. The downstream NRPS module, SfmC, then catalyzes sequential reactions with **86** and **87** to assemble pentacyclic scaffold **93** in a single stroke [97–99]. The reduction (Red) domain at the C-terminus of SfmC reduces the thioester **87** to release aldehyde **88** from SfmB, while the adenylation (A) domain activates **86** and loads it onto the PCP domain of SfmC. The Pictet–Spengler (PS) domain then catalyzes the first diastereoselective PS cyclization to form bicyclic thioester **89** with incorporation of a stereogenic center at C1. The Red domain liberates bicyclic aldehyde **90** by reducing the resulting thioester **89**, while the A domain again activates another molecule of tyrosine derivative **86**, facilitating its loading onto the PCP domain. In the second cycle, the PS domain catalyzes the assembly of the tyrosine derivative and the liberated aldehyde **90** to form tetracyclic thioester **91** bearing an additional chiral center at C11. Subsequent reduction of **91** to aldehyde **92** by the Red domain and spontaneous cyclization would furnish **93**, the pentacyclic core scaffold of **5**. Overall, the single NRPS module SfmC is responsible for the construction of the highly functionalized scaffold **93** from the two simple amino acid derivatives **86** and **88**.

After NRPS-catalyzed scaffold assembly, SfmM1-mediated *N*-methylation at N12, and subsequent SfmO2/O4-promoted oxidation of phenols on A- and E-rings, yield bisquinone **94**. The membrane-bound peptidase SfmE excises the long-chain fatty acid moiety on the N-terminus of the C1 side chain. Secretion through the efflux pump SfmG, and extracellular oxidative deamination catalyzed by the berberine bridge enzyme (BBE)-like enzyme SfmCy2, lead to saframycin S (**95**) [100–101]. To convert the labile hemiaminal moiety at C21 to a more stable aminonitrile, the extracts of the microorganism containing **95** were treated with KCN, allowing the isolation as saframycin A (**5**) [89].

A notable feature of this biosynthetic machinery is the attachment and detachment of the fatty acid moiety. The myristic acid, introduced by SfmA at the beginning, is not attached in the final product **5**. However, detailed investigations by Oikawa and co-workers clearly demonstrated that this fatty acid is essential for SfmC-catalyzed scaffold assembly [97–99]. Furthermore, recent gene disruption studies by Tang and co-workers have also shown that the biosynthetic pathway of safracin, another member of the THIQ family, incorporates palmitic acid (C_16_) at the initial stage of NRPS-catalyzed scaffold assembly [101].

**Scheme 10:**
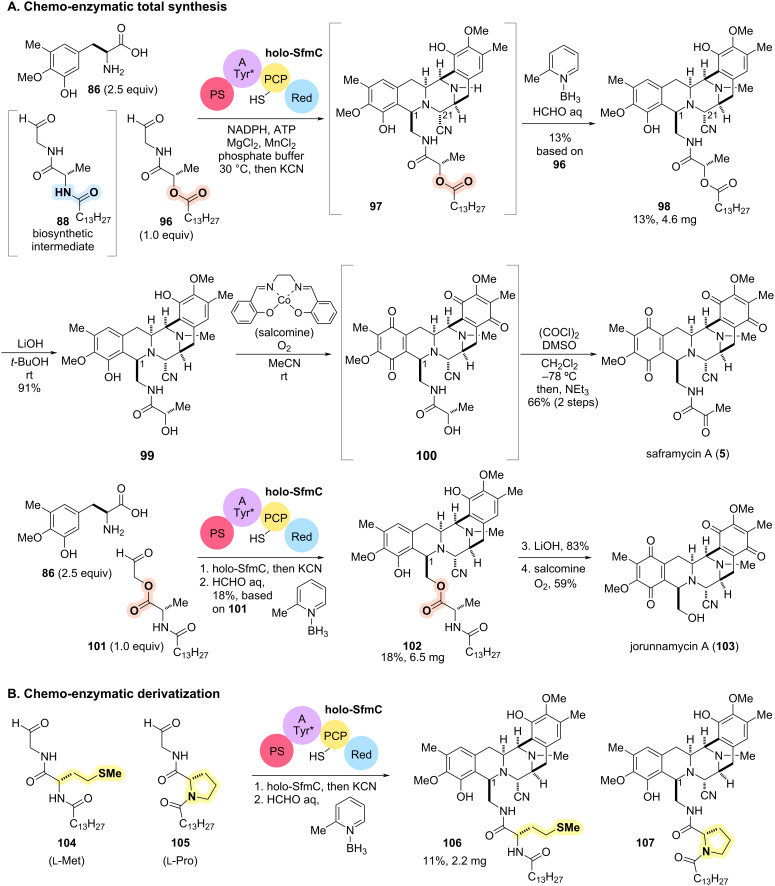
(A) Chemo-enzymatic total synthesis of saframycin A (**5**) and jorunnamycin A (**103**). (B) Chemo-enzymatic structural diversification of the side chain at the C1 position of the bis-THIQ scaffolds.

To achieve the chemo-enzymatic synthesis of the bis-THIQ alkaloid family, Oguri and Oikawa utilized the NRPS module SfmC to construct a highly functionalized scaffold from amino acid derivatives (Scheme 10) [102]. The total synthesis of **5** from original biosynthetic intermediate **93** requires regioselective amide bond cleavage to remove the fatty acid moiety on the C1 side chain (Scheme 9). Although the enzymatic deacylation of **94** can be catalyzed by the membrane-bound peptidase SfmE, similar chemical transformations require harsh conditions and are difficult to perform in the presence of highly reactive functional groups. To circumvent this problem, the research group designed synthetic substrate analogs (**96**, **101**) that mimic the biosynthetic intermediate **88** (Scheme 10A). By replacing an amide linkage in **88** with an ester linkage, the fatty acid side chain could be removed under mild conditions after enzymatic construction of the pentacyclic scaffolds.

The designed and chemically synthesized substrate analog **96** was tolerated by the key NRPS module SfmC despite the structural modification (Scheme 10A). In vitro enzymatic conversion of **96** with tyrosine derivative **86** [103] followed by the addition of KCN concisely furnished the pentacyclic secondary amine **97** bearing the ester linker in the C1 side chain in one pot. After removal of SfmC by precipitation and centrifugation, the reaction mixture containing secondary amine **97** was subjected to the reductive amination using 2-picoline borane as a hydride source, yielding tertiary amine **98** [104]. Without isolation of any intermediate from the simple synthetic substrates **88** and **96**, rapid access to **98** was achieved in 13% yield over two pots based on peptidyl aldehyde **96**. From chemo-enzymatically constructed scaffold **98**, chemical manipulations of functional groups were investigated to achieve the total synthesis of **5** (Scheme 10A). Basic hydrolysis of the ester on the C1 side chain of **98** proceeded under mild conditions and furnished secondary alcohol **99**. Subsequent oxidation of the two phenol rings of **99**, catalyzed by the cobalt complex salcomine, afforded bisquinone **100**. The subsequent Swern oxidation of the resulting secondary alcohol **100** allowed the total synthesis of saframycin A (**5**) in five pots, six steps from the simple synthetic substrates, **86** and **96** [102,105].

By adopting the same strategy to another synthetic substrate analog **101**, the research group also achieved the concise chemo-enzymatic total synthesis of jorunnamycin A (**103**). SfmC-catalyzed enzymatic conversion followed by cyanation and *N*-methylation also converted substrate analog **101** to the corresponding pentacyclic tertiary amine **102** in 18% overall yield based on peptidyl aldehyde **101**. Subsequent simple chemical hydrolysis of the ester and oxidation of the phenol rings allowed concise access to jorunnamycin A (**103**) in just 4 pots from **86** and **101** [102,106]. In subsequent work, Tanifuji and Oguri designed and applied eight variants of the peptidyl aldehyde (e.g., **104**, **105**) bearing various ʟ- and ᴅ-amino acids in place of ʟ-alanine in **88** to the SfmC-catalyzed chemo-enzymatic process [107]. The key enzyme SfmC tolerated all the synthetic analogs of the biosynthetic intermediate **88** and facilitated the rapid synthesis of non-natural bis-THIQ alkaloid-like scaffolds such as **106**, **107** (Scheme 10B).

The essence of this chemo-enzymatic total synthesis was the strategic focus on peptidyl aldehyde **88**, the intermediate that transiently dissociates from the enzymes in the biosynthetic pathway. Leveraging the broad substrate tolerance of SfmC towards **88** facilitated the design of substrate analogs that streamlined enzymatic conversions and subsequent chemical transformations, culminating in the concise total synthesis. This rational and flexible synthetic strategy provided rapid access to the THIQ alkaloid family with a diverse array of side chains by manipulating simple substrate structures. As demonstrated by SfmC, the "catalytic promiscuity" would enhance the utility of enzymes as synthetic tools and facilitate rapid access to a diverse array of natural product analogs through integration with chemical synthesis [108].

## Conclusion

In this review, recent advancements in the field of chemo-enzymatic total synthesis were categorized into three distinct classifications based on the type of enzymatic conversions: 1) regio- and stereoselective late-stage functionalization of core scaffolds, 2) in situ generation of highly reactive intermediates, and 3) one-step construction of macrocyclic or fused multicyclic scaffolds. This classification, along with parallel discussions of the original biosynthetic pathways, helps organize the current state of the art and offers a comprehensive overview of how synthetic methodologies leverage the natural biosynthetic pathways.

The development and optimization of chemo-enzymatic synthetic processes relies heavily on enzyme selection and substrate design. The first strategy, "site- and stereoselective late-stage modification", was highlighted by demonstrating the systematic total synthesis of cotylenol (**1**) and its related natural products, brassicicenes (Scheme 2 and Scheme 3). Natural and engineered P450 enzymes catalyzed the site- and stereoselective oxidative modifications enabling further chemical transformations. Since the scaffolds of terpenes and ribosomally synthesized and post-translationally modified peptides (RiPPs) are first biosynthesized and then modified, the strategy of late-stage enzymatic functionalization of core scaffolds could be effective. Selectivity towards target biomolecules can be tailored by gradually increasing the oxidation level of the complex scaffold or by further site- and stereoselective modifications. Through rational enzyme engineering, the selectivity, efficiency, and robustness of these biocatalysts are expected to enhance their usefulness and generality as synthetic tools.

Apart from oxygenated terpenes and RiPPs, chemo-enzymatic approaches for polyketides and non-ribosomal peptides (NRPs) are considered more suitable for the second and third strategies due to the modular nature of their biosynthetic assembly lines. The second strategy, "in situ generation of highly reactive intermediates", led to the total synthesis of trichodimerol (**2**) and related bisorbicillinoids (Scheme 4). The enzymatic approach, which generates highly reactive, chiral intermediates from stable and achiral compounds, is complementary to chemical methods and is concise. In addition, adjusting the reaction environment with co-solvents and controlling the selectivity of subsequent dimerization may provide an avenue for the future design and application of more sophisticated artificial reaction environments for precise reactivity control.

The third strategy, "regio- and stereoselective scaffold construction", enabled concise access to highly functionalized complex core skeletons of natural products. From the discovery of unknown enzymes to the application of the discovered enzymes to synthetic intermediates, the chemo-enzymatic total synthesis of chalcomoracin (**3**) was comprehensively illustrated (Scheme 5 and Scheme 6). The biosynthetic pathway of natural products in plants still remains challenging due to the complexity and redundancy of their genes, making it difficult to identify the corresponding enzymes. The developed "biosynthetic intermediate probe (BIP)-based target identification” method, a chemical pull-down approach for identifying the target enzymes, would be applied and expanded to the chemo-enzymatic synthesis of other natural products.

In the total synthesis of tylactone (**4**), the combination of two large PKS modules with synthetic substrate mimics facilitated the multistep cascade reaction including carbon chain extension, installation of chiral centers, and macrolactonization in one pot (Scheme 7 and Scheme 8). Moreover, the "site- and stereoselective late-stage modification", presented as the first strategy in this review, was also applied, leading to the systematic chemo-enzymatic synthesis of the related natural products, M-4365 series and juvenimicins. The strategic use of enzymatic and chemical transformations, in accordance with substrate specificity, affords a novel paradigm for exploiting the inherent specificity and efficiency of enzymatic catalysts within synthetic sequences.

The integration of designed substrate analogs that can be transformed by promiscuous enzymes provides a versatile synthetic platform towards natural products and their derivatives. In the total synthesis of saframycin A (**5**) and jorunnamycin A (**103**), the application of a non-standard NRPS module to the synthetic substrate analogs realized the rapid construction of an appropriately functionalized complex scaffold in one pot, with precise control of regio- and diastereoselectivity (Scheme 9 and Scheme 10).

A major limitation of the chemo-enzymatic approach is the current difficulty of designing or evolving enzymes. The chemical synthesis of each substrate and the introduction of mutations into enzymes must be verified to increase their reactivity and selectivity. As the number of applications of chemo-enzymatic hybrid syntheses increases and we better understand the extent to which the structure of enzymes and the reactivity of synthetic substrates can be predicted, guidelines for the rational design of enzymes will likely be established, enabling the rapid identification of optimal substrate–enzyme combinations. The integrative approach of combining intriguing enzymes in ever-evolving biosynthetic research with chemical synthesis, would foster collaborative breakthroughs in the interdisciplinary landscape of natural product biosynthesis, total synthesis as well as function- and diversity-oriented synthesis.

## Data Availability

Data sharing is not applicable as no new data was generated or analyzed in this study.
